# Genomic vulnerability to LINE-1 hypomethylation is a potential determinant of the clinicogenetic features of multiple myeloma

**DOI:** 10.1186/gm402

**Published:** 2012-12-22

**Authors:** Yuka Aoki, Masanori Nojima, Hiromu Suzuki, Hiroshi Yasui, Reo Maruyama, Eiichiro Yamamoto, Masami Ashida, Mitsuhiro Itagaki, Hideki Asaoku, Hiroshi Ikeda, Toshiaki Hayashi, Kohzoh Imai, Mitsuru Mori, Takashi Tokino, Tadao Ishida, Minoru Toyota, Yasuhisa Shinomura

**Affiliations:** 1 First Department of Internal Medicine, Sapporo Medical University School of Medicine, S1, W16, Chuo-Ku, Sapporo 060-8543, Japan; 2 Department of Public Health, Sapporo Medical University School of Medicine, S1, W17, Chuo-ku, Sapporo 060-8556, Japan; 3 Department of Molecular Biology, Sapporo Medical University School of Medicine, S1, W17, Chuo-ku, Sapporo 060-8556, Japan; 4 Department of Regional Health Care and Medicine, Sapporo Medical University School of Medicine, S1, W17, Chuo-ku, Sapporo 060-8556, Japan; 5 Division of Medical Genome Sciences, Research Institute for Frontier Medicine, Sapporo Medical University School of Medicine, S1, W17, Chuo-ku, Sapporo 060-8556, Japan; 6 Department of Hematology, Hiroshima Red Cross and Atomic-bomb Survivors Hospital, 1-9-6 Senda-cho, Hiroshima 730-8619, Japan; 7 Department of Clinical Laboratory, Hiroshima Red Cross and Atomic-bomb Survivors Hospital, 1-9-6 Senda-cho, Naka-ku, Hiroshima 730-8619, Japan; 8 Institute of Medical Science, University of Tokyo, 4-6-1 Shirokanedai, Minato-ku, Tokyo 108-8639, Japan

**Keywords:** Multiple myeloma, Global hypomethylation, Common breakpoints, Repetitive elements, LINE-1

## Abstract

**Background:**

The aim of this study was to clarify the role of global hypomethylation of repetitive elements in determining the genetic and clinical features of multiple myeloma (MM).

**Methods:**

We assessed global methylation levels using four repetitive elements (long interspersed nuclear element-1 (LINE-1), Alu Ya5, Alu Yb8, and Satellite-α) in clinical samples comprising 74 MM samples and 11 benign control samples (7 cases of monoclonal gammopathy of undetermined significance (MGUS) and 4 samples of normal plasma cells (NPC)). We also evaluated copy-number alterations using array-based comparative genomic hybridization, and performed methyl-CpG binding domain sequencing (MBD-seq).

**Results:**

Global levels of the repetitive-element methylation declined with the degree of malignancy of plasma cells (NPC>MGUS>MM), and there was a significant inverse correlation between the degree of genomic loss and the LINE-1 methylation levels. We identified 80 genomic loci as common breakpoints (CBPs) around commonly lost regions, which were significantly associated with increased LINE-1 densities. MBD-seq analysis revealed that average DNA-methylation levels at the CBP loci and relative methylation levels in regions with higher LINE-1 densities also declined during the development of MM. We confirmed that levels of methylation of the 5' untranslated region of respective LINE-1 loci correlated strongly with global LINE-1 methylation levels. Finally, there was a significant association between LINE-1 hypomethylation and poorer overall survival (hazard ratio 2.8, *P *= 0.015).

**Conclusion:**

Global hypomethylation of LINE-1 is associated with the progression of and poorer prognosis for MM, possibly due to frequent copy-number loss.

## Background

Multiple myeloma (MM) is a malignant plasma-cell tumor characterized by various and frequent chromosomal aberrations. Representative examples of these aberrations are loss of chromosome 13, hyperdiploidy, and translocations involving the immunoglobulin heavy chain (IGH) locus situated at 14q32.33. Several studies have shown that these genetic changes are associated with the clinical features of MM, including its prognosis [1-7]. In addition to such genetic changes, recent studies have begun to shed light on the role of epigenetic alterations in the pathogenesis of MM. One of the earliest reports of epigenetic aberrations in MM was of DNA hypermethylation in the promoter CpG islands of p15 and p16 [8-10]. Tumor-specific hypermethylation has also been found in the promoter regions of various tumor suppressors and other tumor-related genes, including *BNIP3*, *DAPK *and *RASD1*, which are associated with prognosis and drug resistance in MM [11-14]. Unexpectedly, however, recent advances in genome-wide analysis revealed that the number of methylated genes declines markedly with the progression of malignant transformation of plasma cells [15,16]. In addition, histone modifications are also involved in the pathogenesis of MM, and are associated with aberrant gene expression or important translocations such as t(4;14) [17,18].

Global DNA hypomethylation is also known to be a common epigenetic alteration in tumor cells [19], and is tightly linked to hypomethylation of DNA repetitive elements [20]. Some repetitive elements, such as long interspersed nuclear element-1 (LINE-1) and Alu, are capable of retrotransposition; that is, they are able to insert themselves into genomic sequences, which can cause genomic instabilities leading to genome-wide mutations, insertions, and deletions [21]. Moreover, because these transpositional activities are usually silenced in association with DNA methylation, global hypomethylation is thought to promote the initiation and progression of tumorigenesis through the aberrant activation of repetitive elements [21]. To date, there have been numerous studies demonstrating hypomethylation of repetitive elements in malignancies [22]. In particular, hypomethylation of LINE-1 is reportedly associated with malignancy, poor prognosis, and chromosomal instability in various types of tumors [23-27].

Our aim in the present study was to clarify the role of global hypomethylation of repetitive elements in determining the genetic and clinical features of MM. To address this issue, we measured the methylation levels of four repetitive elements, and assessed their association with genome-wide copy-number alterations. This integrative analysis of the genetic, epigenetic, and clinical characteristics of MM enabled us to discover a strong association between LINE-1 hypomethylation and copy-number loss and poor prognosis in patients with MM.

## Materials and Methods

### Ethics approval

This study was approved by the institutional review board at Sapporo Medical University (Ethics Committee) and conforms to the tenets of the Declaration of Helsinki. informed consent was obtained prior to sample collection.

### Patients and sample preparation

Bone-marrow aspirates were collected between 2007 and 2010 at the Department of Hematology (Hiroshima Red Cross and Atomic-Bomb Survivors Hospital) and in the 1st Department of Internal Medicine (Sapporo Medical University Hospital) from patients with MM (*n *= 74), patients with monoclonal gammopathy of undetermined significance (MGUS, *n *= 7), and patients with non-plasma-cell tumors with normal plasma cells (NPC, *n *= 4).

We isolated mononuclear cells from the samples using density-gradient separation (Ficoll-Paque; StemCell Technologies Inc., Vancouver, Canada), and then separated the CD138-positive cells using CD138 polymer particles (CD138 MicroBeads; Miltenyi Biotec GmBH, Gladbach, Germany) to isolate the plasma cells. Finally, we extracted the genomic DNA from the CD138-positive cells (QIAamp DNA Blood Mini Kit; Qiagen Inc., Valencia, CA, USA).

### DNA-methylation analysis

Bisulfite conversion of genomic DNA was carried out (EpiTect Bisulfite Kit; Qiagen Inc.). We then used PCR to amplify sequences containing CpG sites in the promoter regions of LINE-1, Alu Yb8, Alu Ya5, and Satellite-α on chromosome 1 (Sat-α), as described previously [27]. The biotinylated PCR products were purified, made single-stranded, and used as templates in a pyrosequencing reaction according to the manufacturer's instructions (Qiagen Inc.). Briefly, the PCR products were bound to streptavidin-conjugated beads (Streptavidin Sepharose HP Beads; Amersham Biosciences Inc., Piscataway, NJ, USA), and were then purified, washed, and denatured with 0.2 mol/L NaOH solution. After addition of 0.3 μmol/L sequencing primer to the purified PCR products, pyrosequencing was carried out using an appropriate system (PSQ96MA) and software (Pyro Q-CpG) (both Biotage AB, Uppsala, Sweden). The primer sequences used in this study are listed in Additional file 1, Table S1.

### Array comparative genomic hybridization

Array comparative genomic hybridization (aCGH) analysis was performed according to the manufacturer's instructions (Agilent Technologies Inc., Wilmington, DE, USA). We first used the restriction enzymes *Alu*I and *Rsa*I to digest 500 ng each of genomic DNA from 67 MM and 6 MGUS samples as well as an aliquot of gender-matched reference DNA (Promega Corp., Madison, WI, USA). We then labeled (Genomic DNA Enzymatic Labeling Kit; Agilent Technologies) the sample and reference DNAs with Cy5 and Cy3, respectively. The labeled DNA was mixed with 25 μg of Cot-1 DNA (Invitrogen), denatured at 95°C for 3 minutes, and incubated at 37°C for 30 minutes. The probe mixture was then hybridized for 40 hours at 65°C (G4450A; SurePrint G3 Human CGH Microarray Kit 8x60K; Agilent Technologies). After washing the array, it was scanned (G2565BA Microarray Scanner; Agilent Technologies) and the fluorescent signals were acquired (Feature Extraction Software; Agilent Technologies). The ADM-2 algorithm included in Genomic Workbench Software (version 6.0; Agilent Technologies) was used to identify copy-number alterations (reference genome: hg 18, threshold = 5.0, minimum number of probes = 3 continuous probes, minimum average of log2 ratio = 0.5). Because the sex chromosomes are strictly controlled through epigenetic mechanisms, they were excluded from this analysis. The Gene Expression Omnibus accession number of the microarray data is GSE33685.

### Methyl-CpG binding domain sequencing

High-throughput sequencing of methylated DNA enriched with methyl-CpG binding domain (MBD) protein (MBD-seq) was performed as follows. Methylated DNA was enriched from 0.5 to 2 µg of genomic DNA obtained from 9 MM, 3 MGUS and 3 NPC specimens (MethylaMiner™Methylated DNA Enrichment Kit; Life Technologies Corp., Carlsbad, CA, USA) according to the manufacturer's instructions. We then prepared a fragment library (SOLiD Fragment Library Construction Kit; Life Technologies) and performed deep sequencing (SOLiD™3 Plus system; Life Technologies). The sequenced reads were mapped onto the human genome (UCSC hg18) using Bowtie software [28].

The number of sequence reads between a pair of sequential aCGH probe sets was counted and then divided by the distance between the probes to obtain the average number of sequence reads per nucleotide. To exclude bias due to intragenomic variation in mapping efficiency, the number of average sequence reads was normalized to the number of sequence reads obtained from control (input) samples to which MBD protein was not applied. We defined that value as the average DNA methylation level. In addition, to exclude bias due to interchromosomal variation caused by copy-number aberrations, the average DNA-methylation levels were normalized to the copy numbers of the respective loci obtained from aCGH. Because the normalized average methylation-level data followed a log-normal distribution, they were log-transposed and statistically standardized.

### Statistical analysis

Differences in mean methylation levels between groups were tested using *t*-tests (for two groups) or ANOVA with a *post hoc *Games-Howell test (for more than two groups). Pearson's correlation coefficients were calculated to evaluate the correlations between two continuous variables. For correlation analysis, log transformation was performed to normalize the number of aCGH probes. Unsupervised hierarchical clustering of the samples using the aCGH results was performed(Cluster 3.0,; originally developed by Michael Eisen, Stanford University). Densities of the repetitive elements were compared using the Mantel-Haenszel test for linear associations. The linear trend of the methylation levels with categorical values were tested using polynomial contrast in general linear models. To evaluate the overall survival (OS) of patients with MM, Kaplan-Meier curves were constructed and evaluated using the log-rank test, and Cox regression was performed. Values of *P*<0.05 were considered statistically significant. Because most variables in this study were inter-associated (e.g., chromosomal aberration status), we did not perform the adjustment for multiple comparisons. All statistical analyses were performed using SPSS Statistics software (version 20 IBM; SPSS Inc., Chicago, IL, USA).

## Results

### Hypomethylation of repetitive elements in MM and MGUS

**Figure 1:**
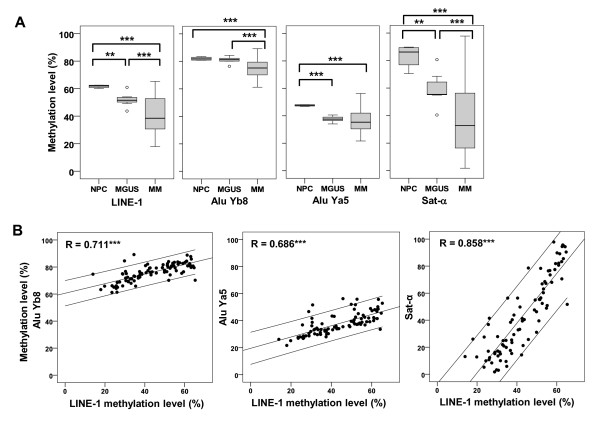
**Quantitative analysis of repetitive-element methylation in malignant melanoma (MM)**. **(A) **Results of quantitative methylation analysis of the indicated repetitive elements in normal plasma cells (NPC; *n *= 4), monoclonal gammopathy of undetermined significance (MGUS; *n *= 7) and MM (*n *= 74). *P < 0.05, **P < 0.01 and ***P<0.001. **(B) **Scatter plots for correlating percentage long interspersed nuclear element-1 (LINE-1) methylation levels with those of the indicated repetitive elements. Pearson's correlation coefficients with regression lines and their 95% confidence intervals are shown on the plots.

The demographic and clinical characteristics of the subjects in this study have been summarized (see Additional file 1, Table S1). We initially performed bisulfite pyrosequencing to assess the methylation levels of four repetitive elements, LINE-1, Alu Yb8, Alu Ya5, and Sat-α, in plasma cells from MM and MGUS samples and from NPC samples (see Additional file 2, Table S2; see Additional file 4, Figure S1). As shown in Figure 1A, the mean levels of repetitive-element methylation were generally lower in MM than in MGUS, and the level in MGUS was lower than in NPC. These observations suggest that repetitive-element methylation declines progressively during tumorigenesis. In addition, we found that there were strong positive correlations between the methylation levels of the four repetitive elements tested (see Additional file 3, Table S3), and that the strongest correlation was between the methylation level of LINE-1 and that of the other three elements (Figure 1B).

### Association between repetitive-element methylation and chromosomal aberrations

**Figure 2:**
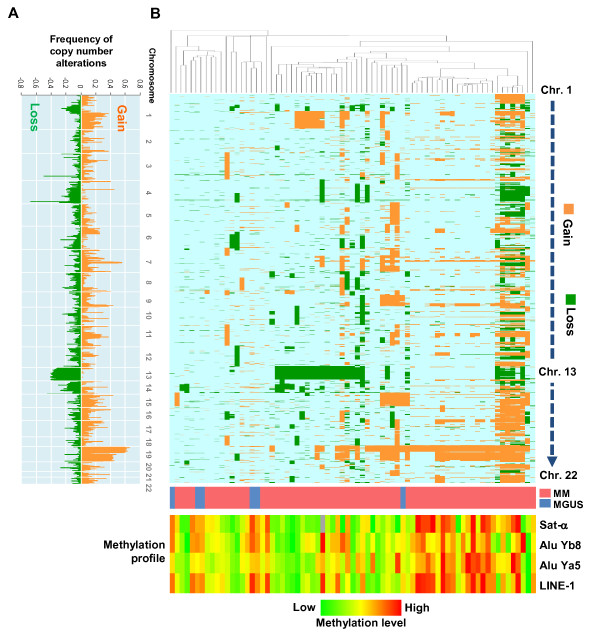
**Genome-wide copy-number analysis in malignant melanoma (MM) and its association with repetitive-element methylation**. **(A) **Summary of the results of array comparative genomic hybridization (aCGH) analyses in MM (*n *= 67) and monoclonal gammopathy of undetermined significance (MGUS; *n *= 6). Loss frequencies(green) are shown on the left, and gain (orange) on the right. **(B) **Unsupervised hierarchical clustering of the MM and MGUS samples using the aCGH data. Color scales represent genomic loss (green), gain (orange) and no change (light blue) in copy number. The heat map shown underneath indicates the methylation levels of the indicated repetitive elements.

We next used aCGH to analyze copy-number alterations in 67 MM and 6 MGUS samples, and assessed their association with the level of repetitive-element methylation (Figure 2A). Consistent with earlier reports, losses on chromosome 13 and gains on chromosome 19 were prevalent among the MM samples (40 to 50%), which confirmed the reliability of our analysis. In addition, loss of the chromosomal arms 1p, 14q, and 22q were also frequently observed in MM. After using unsupervised hierarchical clustering analysis to classify the MM and MGUS samples into several subclasses (Figure 2B), we found that subclasses with prevalent copy-number losses seemed to be associated with hypomethylation of the repetitive elements. By contrast, the MGUS samples and other MM sample subsets were characterized by fewer copy-number alterations and by a lack of repetitive-element hypomethylation (see Figure 2; note the contrast between samples with a blue bar and those with a pink bar in the middle of the figure).

**Figure 3:**
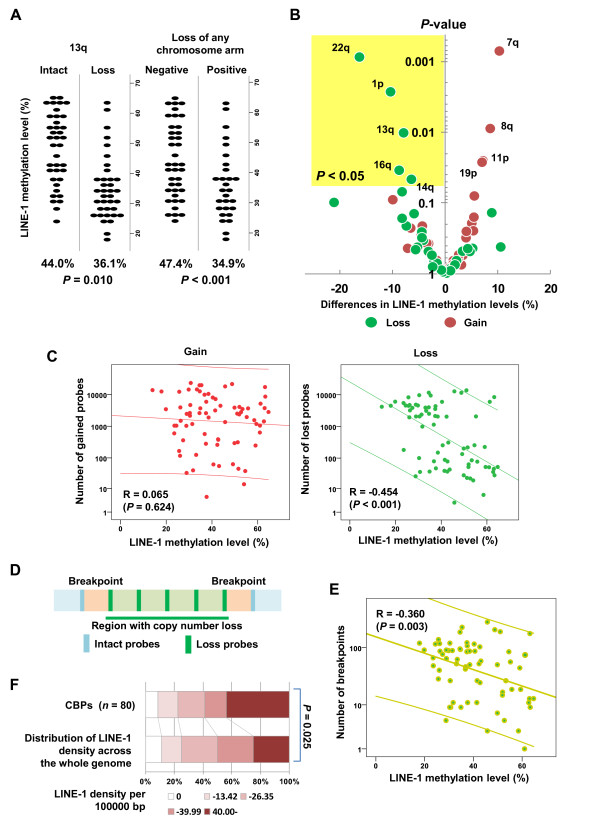
**Association between levels of long interspersed nuclear element-1 (LINE-1) methylation and chromosomal aberrations in malignant melanoma (MM)**. **(A) **comparison of LINE-1 methylation levels between MMs with and without loss of (left) 13q (*n *= 28 and 39, respectively) and (right) any chromosome arm (*n *= 36 and 31, respectively). Each dot represents the level of LINE-1 methylation in a single tumor. The average methylation levels and *P *values are shown underneath. **(B) **Volcano plot showing the relationship between changes in LINE-1 methylation and chromosomal aberrations. Each dot represents a chromosomal arm. Differences in the average levels of LINE-1 methylation between tumors with and without aberrations in the indicated chromosomal arms (loss in green and gain in red) are plotted on the horizontal axis, and *P *values for the respective comparisons are plotted on the vertical axis. Chromosomal arms in which loss exhibited a significant association with LINE-1 hypomethylation (*P *< 0.05) are highlighted. **(C) **Scatter plots showing the correlations between the numbers of array comparative genomic hybridization (aCGH) probes in the gained or lost regions and LINE-1 methylation levels in MMs, with regression lines and their 95% confidence intervals. Note that the numbers of probes in the lost regions showed a significant inverse correlation with the LINE-1 methylation levels. **(D) **Schematic diagram of the putative breakpoints. Green lines indicate aCGH probe sets within lost regions, while blue lines indicate those within intact genomic regions. Light green and light blue areas represent lost and intact genomic regions, respectively. Breakpoints (pink) were defined as regions encompassed by a pair of probe sets located at the boundary of the genomic lost regions. (**E) **Scatter plot showing the correlations between the numbers of breakpoints and LINE-1 methylation levels in MMs, with the regression line and its 95% confidence interval. Note that the numbers of breakpoints showed a significant inverse correlation with the LINE-1 methylation levels. **(F) **Frequencies of the indicated LINE-1 densities (0, 0.01 to 13.43, 13.44 to 26.35, 26.36 to 39.99 and ≥40.00 per 100,000 bp) at the common breakpoints (CBPs, *n *= 80) and across the whole genome. Note that CBPs were significantly associated with higher LINE-1 densities.

We then examined the association between the respective chromosomal aberrations and the levels of repetitive-element methylation. If there were more than 50 probe sets within a copy-number gain or loss region on the same chromosome arm, we defined it as a chromosomal gain or loss, respectively (see Additional file 5, Figure S2A). Using this approach, we found that loss of 13q, which was the most marked chromosomal aberration, frequently coincided with other chromosomal losses (see Additional file 5, Figure S2B). Samples showing a loss of 13q and those with a loss of any chromosomal arm showed significantly lower levels of LINE-1 methylation than those without such losses (Figure 3A). We also found an association between global LINE-1 hypomethylation and gain or loss on the respective chromosomal arms (summarized as a volcano plot in Figure 3B). As highlighted in Figure 3B, we found significant associations between global LINE-1 hypomethylation and loss of 22q, 1p, 16q, and 14q (see Additional file 5, Figure S2C). We also observed a tendency for other chromosomal losses to associate positively with LINE-1 hypomethylation, and similar results were obtained with other repetitive elements (see Additional file 5, Figure S2D).

To quantify the degree of copy-number aberration, we determined for each tumor the number of aCGH probes within copy-number gain or loss regions, and compared that to the level of LINE-1 methylation. In this analysis, the probe number was used as a surrogate for the degree of global genomic alteration. Notably, we found a strong inverse correlation between the numbers of losses found by the probes and the LINE-1 methylation levels, whereas the gains did not show this tendency (Figure 3C). Similar results were obtained for other repetitive elements, but the correlations were weaker than those for LINE-1 (see Additional file 5, Figure S2E).

### LINE-1 density and genomic vulnerability to global hypomethylation

We next focused on the relationship between genomic breakpoints at particular loci and global LINE-1 methylation. Our emphasis on LINE-1 methylation reflected the fact that LINE-1 showed the strongest association with chromosomal and copy-number loss for the repetitive elements we examined in this study. Because the boundaries of regions with copy-number losses are thought to be responsible for particular genomic breaks, we defined breakpoints as regions encompassed by a pair of probe sets located inside and outside the boundary of the loss (Figure 3D). In addition to the number of probes within copy-number loss regions, the total numbers of breakpoints in the respective samples were significantly and inversely correlated (R = -0.360, *P *= 0.003) with the LINE-1 methylation levels (Figure 3E). We also identified 80 common breakpoints (CBPs) present in 4 (5%) of 67 MM cases (Table 1) To assess the relationship between the distribution of LINE-1 and genomic breaks, we used RepeatMasker (Institute for Systems Biology; UCSC Genome Bioinformatics Site [29] to assess LINE-1 density at the respective CBPs. The densities were then categorized into five groups according to their distribution across the entire genome (0, 0.01 to 13.43, 13.44 to 26.35, 26.36 to 39.99, and ≥40.00 per 100,000 bp). Interestingly, the results (summarized in Figure 3F) showed that the average LINE-1 density at CBPs (*n *= 80) was significantly higher than the average density over the entire genome (28.2 in the whole genome vs. 34.4 in the CBPs per 100,000 bp; *P *= 0.025). By contrast, there was no clear correlation between the density of Alu sequences and CBPs (see Additional file 6, Figure S3A).

**Table 1:** List of common breakpoints

**Chr**.	**Locus**	**Start**	**End**	**LINE-1 density** **(per 100,000 bp)**	**Gene**	**Break frequency, %**	**Chr**.	**Locus**	**Start**	**End**	**LINE-1 density** **(per 100000bp**	**Gene**	**Break frequency, %**
1	p34.2	41313735	41355091	58.03	*SCMH1*	10.4	10	q25.1	111391808	111534660	37.1		6.0
1	p34.2	41479154	41520306	21.87		11.9	10	q25.3	116921563	116965495	106.98	*ATRNL1*	10.4
1	p31.3	63102450	63125693	25.81		9.0	10	q25.3	117395507	117462466	37.34	*ATRNL1*	9.0
1	q21.1	145031426	145108225	54.69	*PRKAB2*	6.0	11	p13	36266146	36292214	57.54		6.0
2	p23.2	27659776	28122129	48.23	*BRE*	6.0	11	q23.3	117486844	117510539	84.41	*SCN4B*	14.9
2	p15	63302135	63367996	65.29	*C2orf86*	9.0	12	p13.2	11222379	11312714	55.35	*PRB3*	6.0
2	p11.2	88925032	88984458	53.85		6.0	12	p12.3	15020696	15041656	57.25		7.5
2	q11.2	96394039	96423431	47.63		13.4	12	p11.21	30798016	30861280	41.1		9.0
2	q14.2	118022882	119843354	27.52	*DBI*	7.5	12	q12	34236852	36858944	7.09		6.0
3	p24.2	25637260	25664255	25.93	*TOP2B*	16.4	12	q13.2	53311891	53382664	35.32		16.4
3	p24.2	25799317	25810811	8.7	*OXSM*	10.4	12	q13.2	54253884	54290994	40.42		13.4
3	p24.2	25810870	25997134	29.53		10.4	12	q14.1	56621139	56633663	7.98	*XRCC6BP1*	9.0
3	q11.2	90336752	95063426	1.23		6.0	12	q21.33	90946754	91022602	21.09		7.5
3	q26.1	163874118	163997228	33.3		47.8	13	q14.11	43044213	43114392	45.6	*ENOX1*	11.9
4	q23	99907678	100011201	56.99		6.0	13	q14.11	43114451	43143651	10.27	*ENOX1*	11.9
4	q31.3	152038958	152092912	48.19	*LRBA*	7.5	14	q22.1	48984150	49093570	25.59		6.0
4	q31.3	153097317	153228047	17.59		7.5	14	q31.3	88397409	88411102	51.12	*TTC8*	9.0
4	q34.1	174459916	174490317	23.03	*HMGB2*	6.0	14	q32.33	105080399	105354886	21.86	*IgH*	19.4
5	q13.3	75888651	75947525	25.48	*IQGAP2, F2RL2*	7.5	14	q32.33	105469384	105481523	57.67	*IgH*	11.9
							14	q32.33	105787449	105834932	86.35	*IgH*	9.0
5	q13.3	76284777	76301015	0	*CRHBP*	7.5	14	q32.33	105947052	105977946	61.5	*IgH*	16.4
5	q31.3	140094857	140166875	59.71	*PCDHA1/2/3*	7.5	15	q22.2	59803190	59933103	24.63	*VPS13C*	6.0
5	q33.3	157216334	157301114	41.28		9.0	15	q24.3	74558533	74601045	56.45	*SCAPER*	9.0
5	q34	162798715	162800093	0	*CCNG1*	9.0	16	q11.2	34083801	45122058	2.98	*FLJ43980*	6.0
5	q34	162818326	162833628	19.61	*HMMR*	11.9	17	q12	35221880	35241893	9.99	*IKZF3*	11.9
5	q35.1	170161782	170195254	8.96		7.5	17	q12	35282145	35316098	55.96	*GSDMB*	16.4
5	q35.1	170596026	170659586	33.04	*RANBP17*	6.0	17	q21.2	36666037	36724675	28.99	*KRTAP17-1*	6.0
6	q11.1	62760108	62854732	38.05	*KHDRBS2*	9.0	17	q21.31	38255982	38261676	17.56	*AOC3*	7.5
6	q26	162822332	162896532	9.43	*PARK2*	7.5	17	q25.1	71511380	71527979	0	*EVPL*	6.0
6	q27	166369554	166405962	52.19		7.5	19	q13.12	40328372	40338038	0	*FXYD5*	7.5
7	p15.3	20788779	20857393	14.57		6.0	19	q13.42	61045704	61062298	66.29	*NLRP4*	20.9
7	p14.1	39890729	39952510	58.27		6.0	20	p13	3365914	3411271	59.53	*ATRN*	9.0
7	p14.1	40008069	40034702	45.06	*CDC2L5*	6.0	20	p13	3553255	3588125	43.02	*GFRA4*	9.0
7	q31.2	116015401	116093417	15.38		11.9	20	q12	40298330	40332059	0	*PTPRT*	22.4
7	q35	147108655	147152043	27.66	*CNTNAP2*	6.0	20	q13.12	43765836	43768958	0	*WFDC13*	6.0
8	q12.1	59427474	59488208	41.16	*UBXN2B*	7.5	20	q13.32	57813291	57856200	9.32		9.0
8	q12.1	59519371	59565778	15.08	*CYP7A1*	7.5	20	q13.33	58011342	58074892	33.04	*C20orf197*	7.5
8	q24.3	145297206	145464363	45.47	*BOP1*	13.4	21	q22.11	31050355	31088151	10.58		6.0
9	p23	11563590	11687635	54.01		6.0	22	q11.22	21520273	21588229	38.26	*IgL*	6.0
9	q21.33	87357577	87392381	51.72	*AGTPBP1*	9.0	22	q12.3	34602119	34638273	0	*RBM9*	6.0
10	q25.1	109269991	109444702	43.5		6.0							

long interspersed nuclear element-1 (LINE-1)

In addition, although the involvement of physiological class-switch rearrangements could not be ruled out in our experiments, we noted several CBPs within the IGH locus at 14q32.33, which is known to be a scaffold for distinctive translocations in MM (e.g., t(11;14)(q13;q32) and t(4;14)(p16;q32)). Consistent with the findings described above, we observed significantly greater LINE-1 density in the IGH locus than in the neighboring genomic regions (*P *< 0.001) (see Additional file 6, Figures S3B,C).

### Lower methylation levels at common breakpoints and LINE-1-dense regions in MM

**Figure 4:**
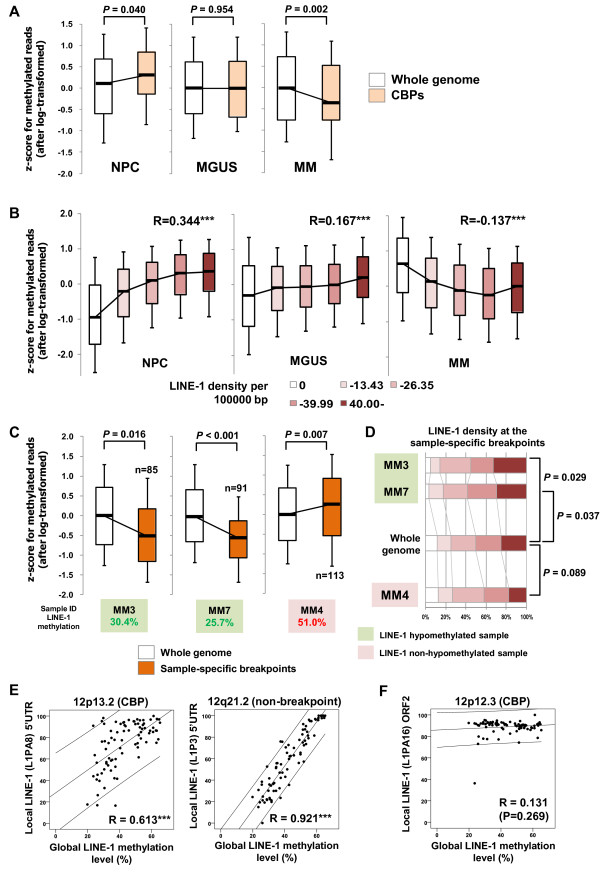
**Association between long interspersed nuclear element-1 (LINE-1) densities and methylation levels in malignant melanoma (MM)**. **(A,B) **Average levels of DNA methylation determined by methyl-CpG binding domain sequencing (MBD-seq) analysis in normal plasma cells (NPC; *n *= 3), monoclonal gammopathy of undetermined significance (MGUS; *n *= 3) and MM (*n *= 9) for **(A) **the whole genome and at the common breakpoints (CBPs, *n *= 80) and **(B) **the respective LINE-density regions. Correlation coefficients are shown above the box plots. ***P < 0.001. Note that average methylation levels were inversely correlated with the LINE-1 densities in MM, while they were positively correlated in NPC. **(C) **Average DNA-methylation levels at the sample-specific breakpoints in representative MM cases. Sample names and global LINE-1 methylation levels are indicated underneath, and the number of breakpoints in each sample is also indicated. Note that LINE-1 methylation levels at sample-specific CBPs were significantly downregulated in samples with global LINE-1 hypomethylation (MM3 and MM7), whereas MM4 exhibited the inverse pattern. **(D) **Frequencies of the indicated LINE-1 densities (0, 0.01 to 13.43, 13.44 to 26.35, 26.36 to 39.99 and ≥40.00 per 100,000 bp) at the sample-specific breakpoints and across the whole genome in three MM cases. Samples with global LINE-1 hypomethylation showed higher LINE-1 densities at the breakpoints (MM3 and MM7), while a sample without global hypomethylation (MM4) did not show that tendency. **(E) **Correlations between the levels of methylation of selected LINE-1 loci and those of global LINE-1 in MM samples (*n *= 73). Methylation in the 5' untranslated region (UTR) of a LINE-1 located at a CBP region on 12p13.2 is shown on the left and that of another LINE-1 located at a non-breakpoint region on 12q21.1 is shown on the right. Pearson's correlation coefficients with regression lines and their 95% confidence intervals are shown on the plots. ***P < 0.001. **(F) **There was no correlation between methylation levels within the gene body region of a selected LINE-1 and global LINE-1 methylation levels in MM samples (*n *= 73). Methylation levels in open reading frame 2 (ORF2) of a LINE-1 located at 12p12.3 were compared with global LINE-1 methylation in MM (*n *= 73). Pearson's correlation coefficient with regression line and its 95% confidence interval are shown on the plot.

To evaluate whole-genome DNA methylation, we next performed MBD-seq for nine MM, three MGUS and three NPC samples [28]. We found that, in MM samples, methylation levels at CBP regions were significantly lower than across the whole genome (Figure 4A). By contrast, we found no such differences in MGUS, while NPC samples showed somewhat higher methylation at CBP regions (Figure 4A). These observations suggest that average DNA-methylation levels at CBP regions decline progressively during tumorigenesis.

We next stratified genomic regions according to their LINE-1 densities, and calculated the average methylation levels in the respective categories. We observed an inverse relationship between methylation levels and LINE-1 densities in MM, whereas methylation levels and LINE-1 densities were positively correlated in MGUS, and this tendency was even clearer in NPC (Figure 4B). These observations again support our hypothesis that methylation levels in LINE-1-enriched regions decline during the development of MM.

When we assessed the methylation levels at breakpoints in respective samples, we observed that all but one MM sample showed reduced methylation at the sample-specific breakpoints, which is not consistent with the observations summarized above (Figure 4C, MM3 and MM7). Interestingly, however, one sample (MM4) showed frequent genomic breaks (133 breakpoints) with higher methylation levels at the breakpoints (Figure 4C). We also found that the sample-specific breakpoints in MM4 were not associated with higher LINE-1 density, which was different from the majority of MM samples (Figure 4D). Collectively, our results suggest that higher LINE-1 densities and hypomethylation are significantly associated with breakpoints in the majority of MM samples, while a subset of samples do not follow this pattern (the small number of exceptional samples are shown in Figure 3C,E).

### Locus-specific LINE-1 methylation correlates with global LINE-1 methylation

To confirm that the global methylation levels determined by our bisulfite pyrosequencing truly reflect the methylation status at the respective loci, we next performed locus-specific bisulfite pyrosequencing at selected LINE-1 loci. We first analyzed the 5' untranslated region (UTR) of a LINE-1 sequence in one of the CBPs on chromosome 12p13.2; genomic alterations are commonly observed in this area in hematological malignancies (see Additional file 7, Figures S4A, B; see Additional file 2, Table S2). In addition, we analyzed a second 5' UTR of a LINE-1 in a non-breakpoint region on chromosome 12q21.1. In both assessments, we observed a significant positive correlation between local and global LINE-1 methylation levels (Figure 4E), and also between the two local LINE-1 methylation levels (see Additional file 7 Figure S4C). By contrast, methylation levels in the gene body region of LINE-1 (ORF2) at 12p12.3 were consistently high in most samples, and did not correlate with global LINE-1 methylation levels (Figure 4F). Because global methylation was also assessed at the 5' UTR of LINE-1 (see Additional file 4, Figure S1A), these results confirmed that global hypomethylation during the development of MM is closely associated with reduced methylation levels at respective LINE-1 loci.

### Association of LINE-1 hypomethylation with a poor prognosis in MM

**Figure 5:**
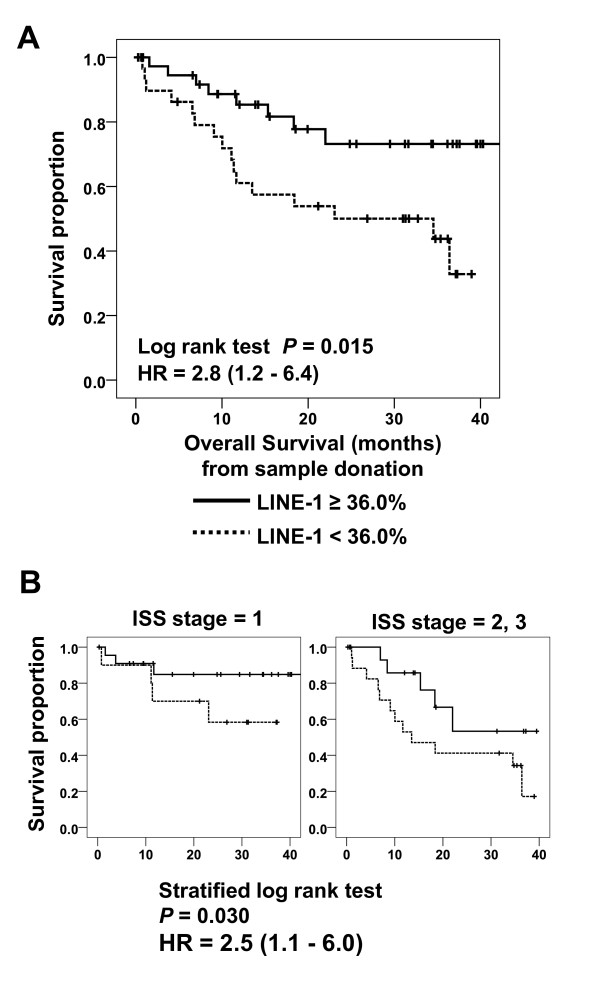
**Association of long interspersed nuclear element-1 (LINE-1) methylation level with prognosis in malignant melanoma (MM)**. **(A, B) **Kaplan-Meier curves for overall survival for patients with MM, with samples stratified based on **(A) **LINE-1 methylation levels and **(B) **International Staging System stage. The *P*-value and hazard ratio (HR) are also shown.

Finally, we examined the association between LINE-1 methylation and prognosis in MM. We first determined the LINE-1 methylation-level that most optimally distinguished between individuals who did and did not survive. We found this to be 36.0%, which was the closest point to the left upper corner of the receiver operating characteristic curve. We then divided all the patients with MM into two groups: those above and below that level. When we compared the survival rates in the two groups, we found that OS from time of sample collection and OS from time of initial diagnosis were both significantly shorter for patients with lower levels of LINE-1 methylation (Figure 5A; see Additional file 8, Figure S5A).

We also found that global LINE-1 methylation level tended to be inversely correlated with stage according to the International Staging System (ISS) (*P *for trend = 0.078; ISS stage 1, 44.7%; stage 2, 41.6%; stage 3, 38.4%). To exclude any effect of different clinical stages for patients, we stratified the samples into two groups: those with ISS stage 1 MM, and those with stage 2 or 3 MM. Even after adjusting for clinical stage, patients with LINE-1 hypomethylation showed significantly poorer OS (Figure 5B). In addition, after adjustment for other prognostic factors, including age, sex, ISS, and loss of chromosome 13 (with stratification by center), LINE-1 hypomethylation was still independently associated with a poor prognosis (hazard ratio = 3.9, *P *= 0.028; Additional file 8, Figure S5B). We also evaluated the associations of Alu Yb8, Alu Ya5, and Sat-α hypomethylation with prognosis, but these were not as strongly associated as LINE-1 hypomethylation (see Additional file 8, Figure S5C). Although a weak association was observed between Alu Ya5 hypomethylation and shorter OS, it was not statistically significant after adjustment for the other prognostic factors (hazard ratio = 1.7, *P *= 0.430).

## Discussion

In this study, we analyzed the DNA methylation of several representative repetitive elements: LINE-1, Alu, and Sat-α. LINE-1 is an abundant retrotransposon that makes up approximately 20% of the mammalian genome. It encodes a reverse transcriptase and is able to amplify and transpose itself within the genome. Alu is one of the short interspersed elements that comprise approximately 10% of the total DNA. Alu does not encode a functional protein, but depends on the machinery of active LINE-1 for transposition [30,31]. Sat-α is a member of the tandemly repeated sequence family, members of which are located at the centromeres of all primate chromosomes [32]. Their presence and spread cause several inherited diseases through the induction of genomic diversity [21,33]. To avoid their inappropriate activation, transcription of repetitive elements is regulated by epigenetic mechanisms, including DNA methylation [21]. Dysregulation of repetitive elements, especially LINE-1, due to hypomethylation, has recently been observed in various tumors [24-27,34-37]. Moreover, insertion of LINE-1 leads to activation of several oncogenes [38,39]. We found strong positive correlations between methylation levels of the all repetitive elements analyzed including local-specific LINE-1 (Figure 1A; see Additional file 3, Table S3, Figure 4E), which suggests the existence of a key factor inducing global hypomethylation of repetitive elements.

Methylation within the promoter regions of protein-coding genes has been found to be generally lower in MM than MGUS or NPC [15,16], and it declines progressively during malignant progression in plasma cells. In the present study, we found a similar decline in the methylation of repetitive elements in MGUS and MM samples. Bollati *et al. *also observed lower levels of repetitive-element methylation in MM, but they did not analyze methylation levels in MGUS [40]. Reduced methylation of repetitive elements has also been reported in precancerous lesions in various organs [41,42], suggesting that global hypomethylation is an early event during tumorigenesis in a number of malignancies. Although methylation of repetitive elements was generally down-regulated in MM compared with NPC, there was substantial case-to-case variability, and the level of methylation, especially of LINE-1, was strongly associated with the degree of copy-number loss and genomic breaks. Similar findings have been reported for other types of malignancy, suggesting that global hypomethylation is associated with chromosomal instability [26,27,35,36]. Consistent with that idea, recent evidence suggests that hypomethylation-induced activation of repetitive elements is directly associated with the chromosomal instability seen in cancer [21,38,39,43,44]. In the context of those earlier reports, our findings indicate that, in malignant cells, LINE-1 may be more active and exhibit a greater potential to induce genomic alterations than other repetitive elements.

We found that LINE-1 density was greater at the 80 CBPs than elsewhere, which suggests that hypomethylation of LINE-1 may be an important factor affecting genomic breaks. For example, the 14q32.33 locus, which exhibits frequent chromosomal translocations and rearrangements in MM and shows very high LINE-1 density, is a site that exhibits possible vulnerability in MM. Conceptually similar to the CBPs described in this study, common fragile sites (CFSs) are highly unstable regions of the genome [45]. Our data indicate that one well-known CFS, *FRA6E/PARK2*, is located at chromosome 6q26 [46], and the surrounding area is also a CBP in MM. A number of CFSs are reportedly located in regions in which there is a high density of both repetitive elements and CBPs [47,48]. In addition, genomic loci at 12p13, where we investigated local LINE-1 methylation levels and which is a CBP, are frequently deleted, and are associated with a poor prognosis in MM [49]. Chromosomal aberrations at 12p13 have also been reported in other types of hematological malignancy [50]. We also identified two CBPs at 13q14, a critical region that is often deleted in MM and other lymphoid disorders, and in which enrichment of LINE-1 and repetitive elements has been reported [51].

Our results demonstrate that DNA-methylation levels at CBPs and regions with high LINE-1 densities decline during the development of MM. Detailed analysis of selected LINE-1 loci revealed that reductions in methylation within the 5' UTR, but not the gene body region, were significantly associated with global hypomethylation. These results indicate that hypomethylation in the 5' UTR of LINE-1 at CBP regions is deeply involved in the development of MM. However, our findings may not support a simple hypothesis that hypomethylation at LINE-1 loci is a determinant of genomic vulnerability at that position, because methylation levels of both CBP-associated and CBP-independent LINE-1 loci positively correlated with global methylation. Collectively, our results suggest that both higher LINE-1 density and hypomethylation in the 5' UTR may be critical factors inducing genomic vulnerability in MM. By contrast, we also observed that a small number of MM cases exhibited frequent genomic breaks, despite relatively high global LINE-1 methylation levels. These cases may be indicative of an underlying mechanism other than LINE-1 hypomethylation, and further study will be needed to understand the complexity of genomic vulnerability in malignant cells.

In addition to LINE-1, Sat-α is reportedly hypomethylated and transcriptionally active in various tumors [23,52]. Although we found the strongest correlation between copy-number aberrations and LINE-1 hypomethylation, further investigation of the significance of other repetitive elements is needed. We observed a stronger association between repetitive-element methylation and chromosomal aberrations than did Bollati *et al. *in their earlier study [40]. This may be attributable to differences in the technologies used to detect copy-number alterations; whereas we performed aCGH that was specialized for comprehensive and sensitive genomic analysis, Bollati *et al. *used fluorescence *in situ *hybridization to detect specific chromosomal aberrations.

Although we demonstrated a novel association between the density and hypomethylation of LINE-1 and genomic alterations in MM, there are several limitations to this study. First, the number of benign control samples was small, and as a result, the statistical power was not sufficient to find significant associations. Second, because we could not obtain control samples of normal DNA from the patients with MM, our results may have been partially influenced by inter-individual copy-number variations. In addition, as described above, we could not rule out the involvement of physiological class-switch rearrangements at the IGH locus. Third, and most importantly, because a longitudinal study was not performed to analyze the molecular changes during the development and progression of MM, the direct causal relationship between LINE-1 hypomethylation and genomic vulnerability remains to be validated in a future functional study.

Despite the aforementioned limitations, we found that LINE-1 hypomethylation is associated with a poor prognosis in MM. Even after stratification and adjustment for several confounders, the association remained statistically significant, suggesting LINE-1 hypomethylation as an independent prognostic factor. Moreover, our findings are consistent with similar results obtained in other malignancies, and is supported by several reports in which a poorer prognosis was observed in MM with non-hyperdiploidy [23,25,36,37].. Our data suggest that non-hyperdiploidy, which is indicative of genomic and chromosomal loss, is associated with LINE-1 hypomethylation [3].

## Conclusions

Our findings suggest that global hypomethylation of repetitive elements may increase the malignant potential of myeloma cells by inducing broad copy-number losses. In particular, LINE-1 is a probable contributing factor for chromosomal aberrations and the progression of MM under conditions of global hypomethylation. Our results also indicate that clinical management should include analysis of repetitive-element methylation. For further investigation, we plan a detailed functional study to clarify the cause of global hypomethylation and the precise mechanism of hypomethylation-mediated genomic breaks in MM.

## Competing interests

The authors declare that they have no competing interests.

## Authors' contributions

Contribution: YA designed research, performed experiments, analyzed data and wrote the paper; MN designed and organized research, analyzed data and wrote the paper; HS designed research and wrote the paper; HY and MI provided biomaterial and analyzed data; RM analyzed data; EY, KI, and YI designed research; MA performed experiments; AH, HI, and TH, and TI provided biomaterial; MM and TT analyzed data; and MT planned and organized research. All authors read and approved the final manuscript.

## Supplementary Material

Additional file 1 **Table S1**. Demographic and clinical characteristics of the subjects in this study. Click here for file

Additional file 2 **Table S2**. Primer information. Click here for file

Additional file 3 **Table S3**. Comparison of the methylation levels and correlation of the repetitive elements. Click here for file

Additional file 4 **Figure S1**. Schematic representations of the repetitive elements and CpG sites analyzed in this study. Regions amplified by PCR and analyzed by pyrosequencing are shown underneath the structures. Click here for file

Additional file 5 **Figure S2**. **(A) **Summary of probe numbers included in gain/loss regions in each chromosome arm of malignant melanoma (MM) cases. The X-axis represents the probe number and the Y-axis represents the frequency. The bimodal distribution pattern indicates that chromosome arms are largely divided into two groups, those with a smaller number of aberrations (less than 50 probes) and those with a larger number of aberrations (more than 50 probes). **(B) **Summary of chromosomal losses (green) in MM (*n *= 67); note that the majority of MMs showing any chromosomal loss showed a loss of 13q. **(C) **Comparisons of long interspersed nuclear element-1 (LINE-1) methylation levels between MMs with and without loss of 1p, 14q, or 16q. **(D) **Volcano plots showing the relationship between changes in the methylation of the indicated repetitive elements and chromosomal aberrations. Each dot represents a chromosomal arm, and differences in the average methylation levels between tumors with and without aberrations (losses are in green, gains are in red) in the arms of interest are plotted on the horizontal axis, with *P *values plotted on the vertical axis. **(E) **Scatter plots showing the correlations between the numbers of array comparative genomic hybridization (aCGH) probes in the gain/loss regions and the levels of methylation of the indicated repetitive elements. Note that for all of the repetitive elements analyzed, the degree of deletion inversely correlated with methylation level. Click here for file

Additional file 6 **Figure S3**. (**A) **Frequencies of the indicated Alu densities (0, 0.01 to 13.43, 13.44 to 26.35, 26.36 to 39.99 and ≥40.00 per 100,000 bp) in the whole genome and common breakpoints (CBPs, *n *= 80). Note that CBPs were not significantly associated with Alu densities (*P *= 0.254). **(B) **Frequencies of the respective long interspersed nuclear element-1 (LINE-1) densities in the immunoglobulin heavy chain (IGH) locus and other loci at 14q32,33 (***P < 0.001). **(C) **Schematic representation of the 14q32.33 region. LINE-1 densities are shown on the top, and the genes are indicated on the bottom. Click here for file

Additional file 7 **Figure S4**. Analysis of methylation in selected long interspersed nuclear element-1 (LINE-1) loci in malignant melanoma (MM). **(A) **Summarized results of array comparative genomic hybridization (aCGH) on chromosome 12 in MM samples (*n *= 12). Losses are indicated in green, and common breakpoints (CBPs) at 12p13.3 and 12p12.3 are indicated by red arrows. **(B) **Locations of primers used in the locus-specific bisulfite pyrosequencing; shown are original (not bisulfite-converted) sequences. A non-CBP LINE-1 and two CBP-associated LINE-1 loci were selected and analyzed. Forward primers were located outside the LINE-1 sequences so that only unique sequences were amplified by PCR. **(C) **Correlation between the methylation levels of the 5' untranslated regions (UTRs) of two local LINE-1s. Pearson's correlation coefficients with the regression line and its 95% confidence interval are shown on the plot. Click here for file

Additional file 8 **Figure S5**. **(A,B) **Kaplan-Meier curves for overall survival from time of: **(A) **initial diagnosis for patients with MM stratified based on long interspersed nuclear element-1 (LINE-1) methylation levels; **(B) **sample collection for patients with MM after stratification based on the presence or absence of 13q deletion; and (C) initial diagnosis for patients with MM stratified based on the levels of methylation of the indicated repetitive elements. Click here for file
