# Correction: Genomic vulnerability to LINE-1 hypomethylation is a potential determinant of the clinicogenetic features of multiple myeloma

**DOI:** 10.1186/gm491

**Published:** 2013-10-11

**Authors:** Yuka Aoki, Masanori Nojima, Hiromu Suzuki, Hiroshi Yasui, Reo Maruyama, Eiichiro Yamamoto, Masami Ashida, Mitsuhiro Itagaki, Hideki Asaoku, Hiroshi Ikeda, Toshiaki Hayashi, Kohzoh Imai, Mitsuru Mori, Takashi Tokino, Tadao Ishida, Minoru Toyota, Yasuhisa Shinomura

**Affiliations:** 1 First Department of Internal Medicine, Sapporo Medical University School of Medicine, S1, W16, Chuo-Ku, Sapporo 060-8543, Japan; 2 Department of Public Health, Sapporo Medical University School of Medicine, S1, W17, Chuo-ku, Sapporo 060-8556, Japan; 3 Department of Molecular Biology, Sapporo Medical University School of Medicine, S1, W17, Chuo-ku, Sapporo 060-8556, Japan; 4 Department of Regional Health Care and Medicine, Sapporo Medical University School of Medicine, S1, W17, Chuo-ku, Sapporo 060-8556, Japan; 5 Division of Medical Genome Sciences, Research Institute for Frontier Medicine, Sapporo Medical University School of Medicine, S1, W17, Chuo-ku, Sapporo 060-8556, Japan; 6 Department of Hematology, Hiroshima Red Cross and Atomic-bomb Survivors Hospital, 1-9-6 Senda-cho, Hiroshima 730-8619, Japan; 7 Department of Clinical Laboratory, Hiroshima Red Cross and Atomic-bomb Survivors Hospital, 1-9-6 Senda-cho, Naka-ku, Hiroshima 730-8619, Japan; 8 Institute of Medical Science, University of Tokyo, 4-6-1 Shirokanedai, Minato-ku, Tokyo 108-8639, Japan

## Correction

An error was made in the figure and supplementary file legends for this study [1]. The figure legend titles should read as follows:

Figure 1. Quantitative analysis of repetitive-element methylation in multiple myeloma (MM).

Figure 2. Genome-wide copy-number analysis in multiple myeloma (MM) and its association with repetitive-element methylation.

Figure 3. Association between levels of long interspersed nuclear element-1 (LINE-1) methylation and chromosomal aberrations in multiple myeloma (MM).

Figure 4. Association between long interspersed nuclear element-1 (LINE-1) densities and methylation levels in multiple myeloma (MM).

Figure 5. Association of long interspersed nuclear element-1 (LINE-1) methylation level with prognosis in multiple myeloma (MM).

The additional file legends should read as follows:

**Additional file 5. Figure S2.** (A) Summary of probe numbers included in gain/loss regions in each chromosome arm of multiple myeloma (MM) cases. The X-axis represents the probe number and the Y-axis represents the frequency. The bimodal distribution pattern indicates that chromosome arms are largely divided into two groups, those with a smaller number of aberrations (less than 50 probes) and those with a larger number of aberrations (more than 50 probes). (B) Summary of chromosomal losses (green) in MM (n = 67); note that the majority of MMs showing any chromosomal loss showed a loss of 13q. (C) Comparisons of long interspersed nuclear element-1 (LINE-1) methylation levels between MMs with and without loss of 1p, 14q, or 16q. (D) Volcano plots showing the relationship between changes in the methylation of the indicated repetitive elements and chromosomal aberrations. Each dot represents a chromosomal arm, and differences in the average methylation levels between tumors with and without aberrations (losses are in green, gains are in red) in the arms of interest are plotted on the horizontal axis, with P values plotted on the vertical axis. (E) Scatter plots showing the correlations between the numbers of array comparative genomic hybridization (aCGH) probes in the gain/loss regions and the levels of methylation of the indicated repetitive elements. Note that for all of the repetitive elements analyzed, the degree of deletion inversely correlated with methylation level.

**Additional file 7. Figure S4.** Analysis of methylation in selected long interspersed nuclear element-1 (LINE-1) loci in multiple myeloma (MM). (A) Summarized results of array comparative genomic hybridization (aCGH) on chromosome 12 in MM samples (n = 12). Losses are indicated in green, and common breakpoints (CBPs) at 12p13.3 and 12p12.3 are indicated by red arrows. (B) Locations of primers used in the locus-specific bisulfite pyrosequencing; shown are original (not bisulfite-converted) sequences. A non-CBP LINE-1 and two CBP-associated LINE-1 loci were selected and analyzed. Forward primers were located outside the LINE-1 sequences so that only unique sequences were amplified by PCR. (C) Correlation between the methylation levels of the 5′ untranslated regions (UTRs) of two local LINE-1 s. Pearson’s correlation coefficients with the regression line and its 95% confidence interval are shown on the plot.

## References

[B1] Aoki Y Nojima M Suzuki H Yasui H Maruyama R Yamamoto E Ashida M Itagaki M Asaoku H Ikeda H Hayashi T Imai K Mori M Tokino T Ishida T Toyota M Shinomura Y Genomic vulnerability to LINE-1 hypomethylation is a potential determinant of the clinicogenetic features of multiple myeloma Genome Medicine 2012 5 101 10.1186/gm402 23259664 PMC4064317

