# Supplementary material for: Genomic vulnerability to LINE-1 hypomethylation is a potential determinant of the clinicogenetic features of multiple myeloma
Source: Genome Med. 2012 Dec 22;4(12):101. doi: 10.1186/gm402 (PMC4064317; doi:10.1186/gm402)
Supplement: Additional file 1 — Table S1. Demographic and clinical characteristics of the subjects in this study. [file gm402-S1.DOCX]

| **Table S1**. Demographic and clinical characteristics of the subjects in this study | | | | |
| --- | --- | --- | --- | --- |
|  |  |  |  |  |
|  |  | MGUS | MM |  |
| *n* |  | 7 | 74 |  |
| Sex (male / female) |  | 4 / 3 | 34 / 40 |  |
| Age (range) |  | 68 (52-93) | 67 (45-93) |  |
|  |  |  |  |  |
| Type | IgG-κ | 4 | 25 |  |
|  | IgG-λ | 2 | 16 |  |
|  | IgA-κ | - | 8 |  |
|  | IgA-λ | - | 6 |  |
|  | BJP | - | 10 |  |
|  | Other or unknown | 1 | 9 |  |
|  |  |  |  |  |
| ISS | I | N/A | 35 |  |
|  | II |  | 12 |  |
|  | III |  | 24 |  |
|  | unknown |  | 3 |  |
|  |  |  |  |  |
| Prognosis | Alive | 7 | 44 |  |
|  | Dead | - | 25 |  |
|  | Unknown | - | 5 |  |
|  |  |  |  |  |
| MM: multiple myeloma, N/A: not available | | |  |  |
