# Supplementary material for: Genomic vulnerability to LINE-1 hypomethylation is a potential determinant of the clinicogenetic features of multiple myeloma
Source: Genome Med. 2012 Dec 22;4(12):101. doi: 10.1186/gm402 (PMC4064317; doi:10.1186/gm402)
Supplement: Additional file 2 — Table S2. Primer information. [file gm402-S2.DOCX]

| **Table S2.** Primer information | | |  |
| --- | --- | --- | --- |
|  |  |  |  |
| Elements | Class | PCR primer sequence |  |
| LINE-1 (L1HS) | L1HS | F:5'-TTTTGAGTTAGGTGTGGGATATAGTT-3' |  |
|  |  | R:5'-CAAAAAATCAAAAAATTCCCTTTCC-3' |  |
| Alu | Yb8 | F:5'-YGTAATTTYGGTTTATTGTAAGTTT-3' |  |
|  |  | R:5'-ATCCTAACTAACAAAATAAAACCC-3' |  |
| Alu | Ya5 | F:5'-GAGGTTGAGGTAGGAGAA-3' |  |
|  |  | R:5'-CCCAAACTAAAATACAATAAC-3 |  |
| Sat-α |  | F:5'-AGTTTAATTTATAGAGTAGAGTAG-3' |  |
|  |  | R:5'-AAATCTTCACTTACAAATACCAC-3' |  |
| Non-CBP LINE-1 (5’UTR) | L1P3 | F-5’-GTTGGATTATTGGTTATTTAAAATTGA-3’ |  |
|  |  | R-5’-ATTCCCTAACCCCCTTACCC-3’ |  |
| CBP LINE-1 (5’UTR) | L1PA8 | F-5’-AAATTGTAGGGTTTGGGAAAGT-3’ |  |
|  |  | R-5’-AACACAATCTATCATAACTTCCCTTT-3’ |  |
| CBP LINE-1 (ORF2) | L1PA16 | F-5’-GAAGTATTGGTTTGTTTTATATTTGTGG-3’ |  |
|  |  | R-5’-TTTCTCCATTATTTATTTTTATCAACT-3’ |  |
|  |  | Y: C or T |  |
|  |  |  |  |
| Elements |  | Pyrosequencing primer / sequence to analyze | No. of CpG analyzed |
| LINE-1 (L1HS) | L1HS | 5'-GGGTGGGAGTGAT-3'/ | 3 |
|  |  | T**CG**ATTTTTTAGGTG**CG**TT**CG** |  |
| Alu Yb8 | Yb8 | 5'-TTAGTAGTTGGGATTATAGG-3'/ | 5 |
|  |  | **CG**TT**CG**TTAT**CGCG**TT**CG** |  |
| Alu Ya5 | Ya5 | 5'-GTTGAGGTAGGAGAA-3'/ | 3 |
|  |  | TGG**CG**TGAATT**CG**GGAGG**CG** |  |
| Sat-α |  | 5'-TGGGATTTTTTTGAGAATTT-3'/ | 2 |
|  |  | TTGGAAA**CG**GGATAAATTTTA**CG** |  |
| Non-CBP LINE-1 (5’UTR) | L1P3 | 5’-GGTGAGTCGAAGTAGGGTGGGGTAG-3’/ | 2 |
|  |  | GTAT**CG**TTTTGTT**CG** |  |
| CBP LINE-1 (5’UTR) | L1PA8 | 5’-GGAGGATAAGTAGAAGTAAGGTAGG^3’/ | 2 |
|  |  | GTGT**CG**TTTTATTT**CG** |  |
| CBP LINE-1 (ORF2) | L1PA16 | 5’-AAATAGTATGGTATAAAAATAGATATATAG-3’/ | 2 |
|  |  | AT**CG**GTGGAATAGAATAGGGAA**CG** |  |
