# Supplementary material for: Genomic vulnerability to LINE-1 hypomethylation is a potential determinant of the clinicogenetic features of multiple myeloma
Source: Genome Med. 2012 Dec 22;4(12):101. doi: 10.1186/gm402 (PMC4064317; doi:10.1186/gm402)
Supplement: Additional file 3 — Table S3. Comparison of the methylation levels and correlation of the repetitive elements. [file gm402-S3.DOCX]

| **Table S3.** Comparison of the methylation levels and correlation of the repetitive elements | | | | | | | | | | | | | | | | |
| --- | --- | --- | --- | --- | --- | --- | --- | --- | --- | --- | --- | --- | --- | --- | --- | --- |
|  |  |  |  |  |  |  |  |  |  |  |  |  |  |  |  |  |
|  | Methylation levels (%) | | | | | | | | | | |  |  |  |  |  |
|  | NPC | | | |  | MGUS | | |  | MM | |  | Correlation coefficients* | | | |
|  | Mean | SD | *P* value† (vs. MGUS) | *P* value† (vs. MM) |  | Mean | SD | *P* value† (vs. MM) |  | Mean | SD |  | LINE-1 | Alu Yb8 | Alu Ya5 | Sat-α |
| LINE-1 | 61.7 | 1.1 | 0.005 | <0.001 |  | 52.0 | 5.2 | 0.002 |  | 41.7 | 13.0 |  | - | 0.686 | 0.711 | 0.858 |
| Alu Yb8 | 81.9 | 1.2 | 0.676 | <0.001 |  | 81.0 | 2.5 | <0.001 |  | 75.0 | 6.5 |  | 0.711 | - | 0.516 | 0.542 |
| Alu Ya5 | 47.6 | 0.6 | <0.001 | <0.001 |  | 37.6 | 2.3 | 0.676 |  | 37.1 | 8.1 |  | 0.686 | 0.516 | - | 0.588 |
| SatA | 83.2 | 8.9 | 0.015 | <0.001 |  | 59.4 | 12.6 | 0.013 |  | 39.8 | 27.8 |  | 0.858 | 0.588 | 0.542 | - |
|  |  |  |  |  |  |  |  |  |  |  |  |  |  |  |  |  |
| NPC: non-plasma cell, MM: multiple myeloma, SD: standard deviation | | | | | | | | |  |  |  |  |  |  |  |  |
| * *P* < 0.001 for all correlation coefficients | | | | |  |  |  |  |  |  |  |  |  |  |  |  |
| † Multiple comparisons by Games–Howell test | | | | |  |  |  |  |  |  |  |  |  |  |  |  |
