# Supplementary material for: Genomic vulnerability to LINE-1 hypomethylation is a potential determinant of the clinicogenetic features of multiple myeloma
Source: Genome Med. 2012 Dec 22;4(12):101. doi: 10.1186/gm402 (PMC4064317; doi:10.1186/gm402)
Supplement: Additional file 4 — Figure S1. Schematic representations of the repetitive elements and CpG sites analyzed in this study. Regions amplified by PCR and analyzed by pyrosequencing are shown underneath the structures. [file gm402-S4.PPT]

## Slide 1
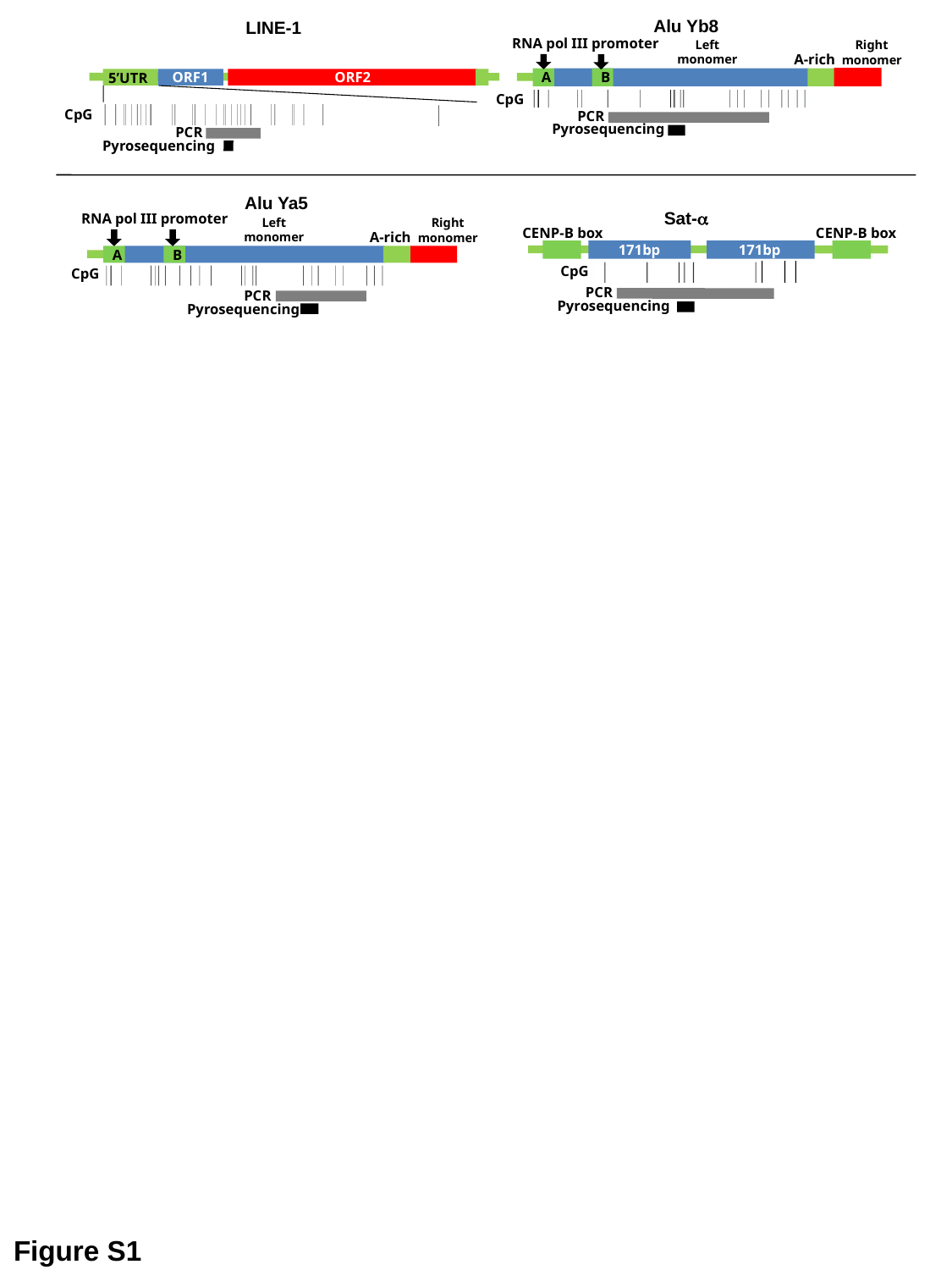

Alu Yb8
LINE-1
RNA pol III promoter
Left monomer
Right monomer
A
B
CpG
PCR
Pyrosequencing
A-rich
5’UTR
ORF1
ORF2
CpG
PCR
Pyrosequencing
Alu Ya5
Sat-α
RNA pol III promoter
Left monomer
Right monomer
A
B
CpG
PCR
Pyrosequencing
CENP-B box
CENP-B box
171bp
171bp
CpG
PCR
Pyrosequencing
A-rich
Figure S1
