# Supplementary figures and images for: Genomic vulnerability to LINE-1 hypomethylation is a potential determinant of the clinicogenetic features of multiple myeloma
Source: Genome Med. 2012 Dec 22;4(12):101. doi: 10.1186/gm402 (PMC4064317; doi:10.1186/gm402)

## Slide 1
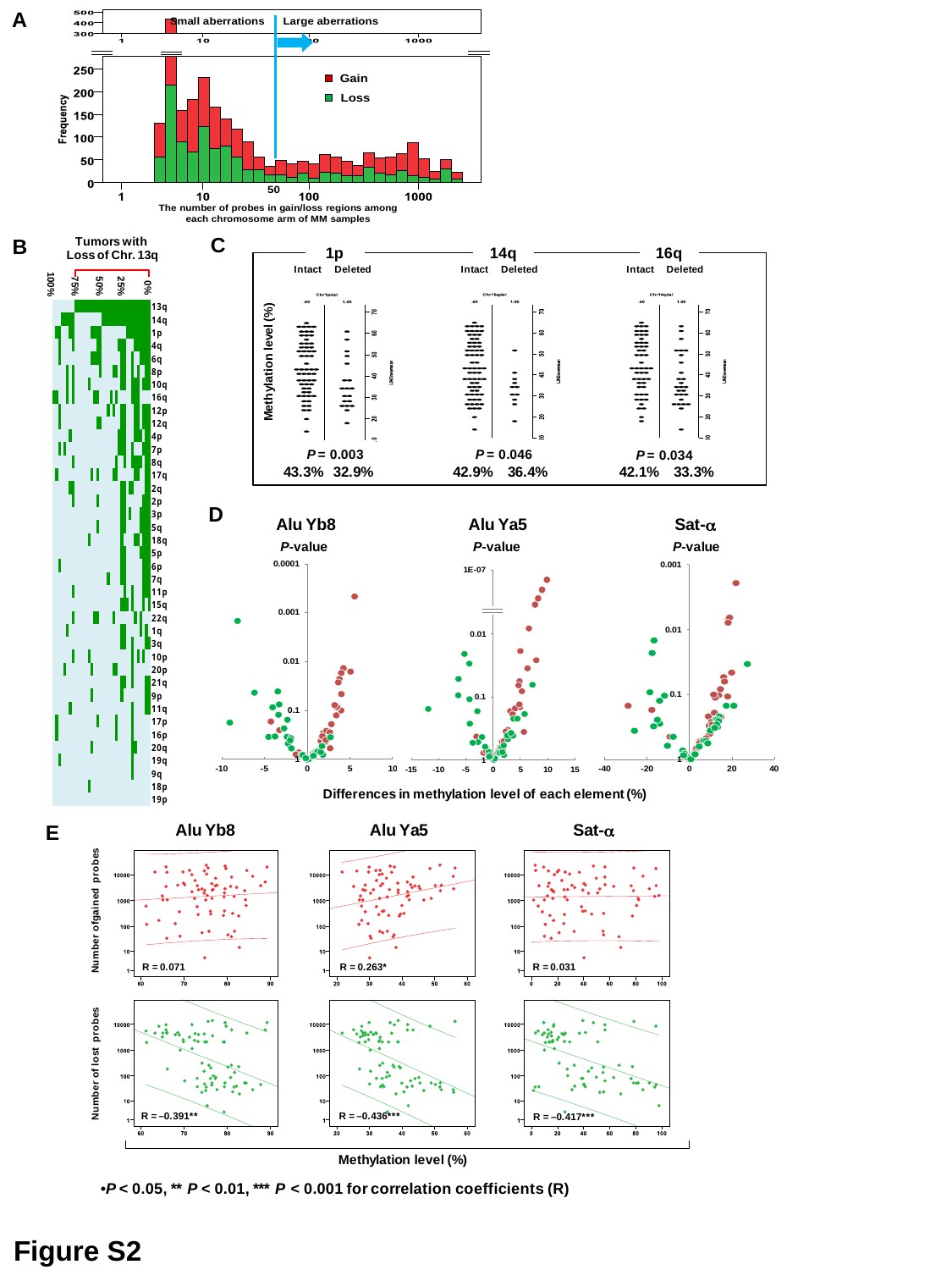

A
Figure S2

Supplement: Additional file 5 — Figure S2. (A) Summary of probe numbers included in gain/loss regions in each chromosome arm of malignant melanoma (MM) cases. The X-axis represents the probe number and the Y-axis represents the frequency. The bimodal distribution pattern indicates that chromosome arms are largely divided into two groups, those with a smaller number of aberrations (less than 50 probes) and those with a larger number of aberrations (more than 50 probes). (B) Summary of chromosomal losses (green) in MM (n = 67); note that the majority of MMs showing any chromosomal loss showed a loss of 13q. (C) Comparisons of long interspersed nuclear element-1 (LINE-1) methylation levels between MMs with and without loss of 1p, 14q, or 16q. (D) Volcano plots showing the relationship between changes in the methylation of the indicated repetitive elements and chromosomal aberrations. Each dot represents a chromosomal arm, and differences in the average methylation levels between tumors with and without aberrations (losses are in green, gains are in red) in the arms of interest are plotted on the horizontal axis, with P values plotted on the vertical axis. (E) Scatter plots showing the correlations between the numbers of array comparative genomic hybridization (aCGH) probes in the gain/loss regions and the levels of methylation of the indicated repetitive elements. Note that for all of the repetitive elements analyzed, the degree of deletion inversely correlated with methylation level. [file gm402-S5.PPT]
