# Supplementary material for: Genomic vulnerability to LINE-1 hypomethylation is a potential determinant of the clinicogenetic features of multiple myeloma
Source: Genome Med. 2012 Dec 22;4(12):101. doi: 10.1186/gm402 (PMC4064317; doi:10.1186/gm402)
Supplement: Additional file 6 — Figure S3. (A) Frequencies of the indicated Alu densities (0, 0.01 to 13.43, 13.44 to 26.35, 26.36 to 39.99 and ≥40.00 per 100,000 bp) in the whole genome and common breakpoints (CBPs, n = 80). Note that CBPs were not significantly associated with Alu densities (P = 0.254). (B) Frequencies of the respective long interspersed nuclear element-1 (LINE-1) densities in the immunoglobulin heavy chain (IGH) locus and other loci at 14q32,33 (***P < 0.001). (C) Schematic representation of the 14q32.33 region. LINE-1 densities are shown on the top, and the genes are indicated on the bottom. [file gm402-S6.PPT]

## Slide 1
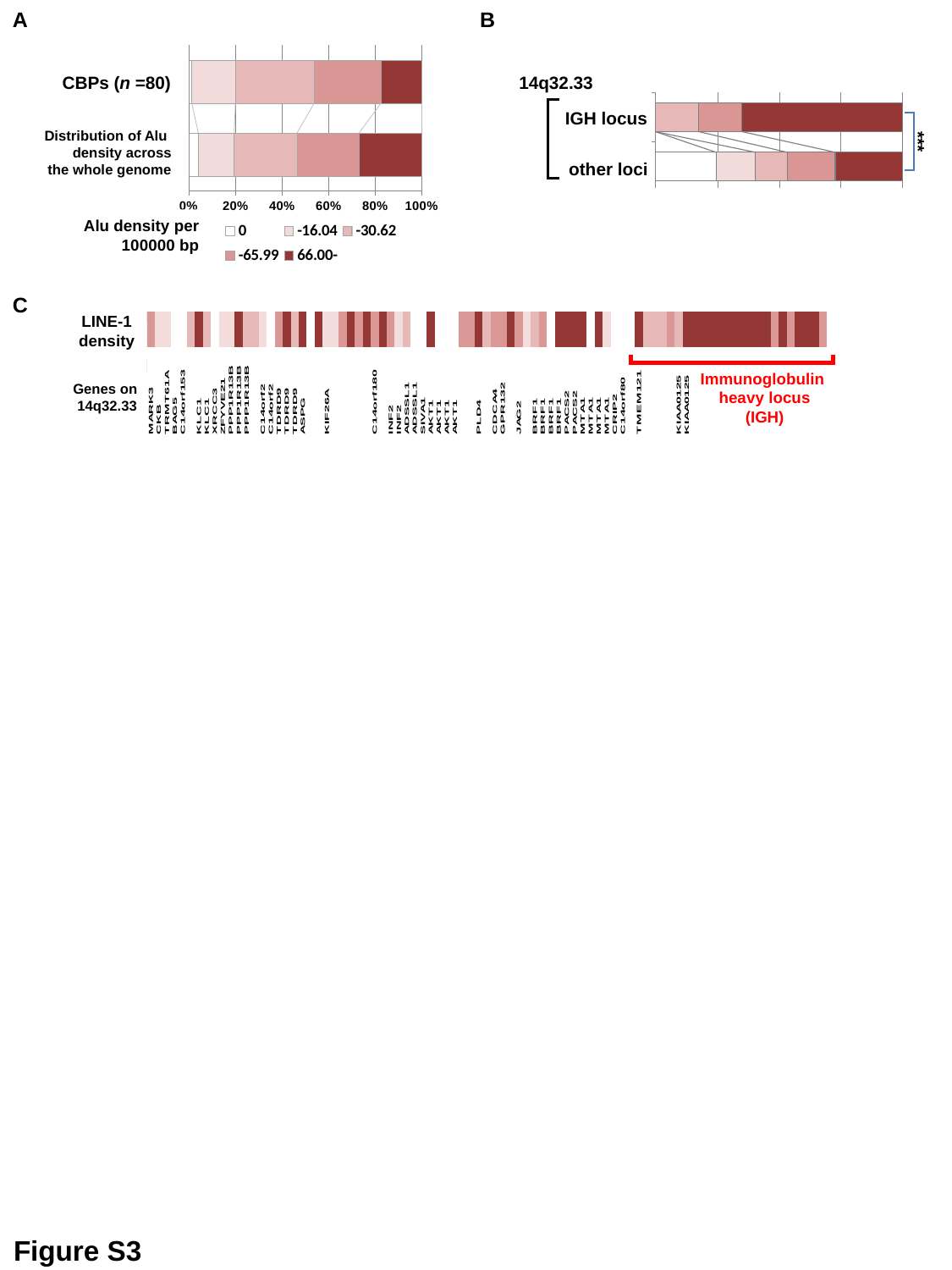

A
B
CBPs (n =80)
14q32.33
 IGH locus
***
other loci
Distribution of Alu density across
the whole genome
Alu density per 100000 bp
C
LINE-1 density
Immunoglobulin
heavy locus
(IGH)
Genes on
 14q32.33
Figure S3
