# Supplementary material for: Genomic vulnerability to LINE-1 hypomethylation is a potential determinant of the clinicogenetic features of multiple myeloma
Source: Genome Med. 2012 Dec 22;4(12):101. doi: 10.1186/gm402 (PMC4064317; doi:10.1186/gm402)
Supplement: Additional file 7 — Figure S4. Analysis of methylation in selected long interspersed nuclear element-1 (LINE-1) loci in malignant melanoma (MM). (A) Summarized results of array comparative genomic hybridization (aCGH) on chromosome 12 in MM samples (n = 12). Losses are indicated in green, and common breakpoints (CBPs) at 12p13.3 and 12p12.3 are indicated by red arrows. (B) Locations of primers used in the locus-specific bisulfite pyrosequencing; shown are original (not bisulfite-converted) sequences. A non-CBP LINE-1 and two CBP-associated LINE-1 loci were selected and analyzed. Forward primers were located outside the LINE-1 sequences so that only unique sequences were amplified by PCR. (C) Correlation between the methylation levels of the 5' untranslated regions (UTRs) of two local LINE-1s. Pearson's correlation coefficients with the regression line and its 95% confidence interval are shown on the plot. [file gm402-S7.PPT]

## Slide 1
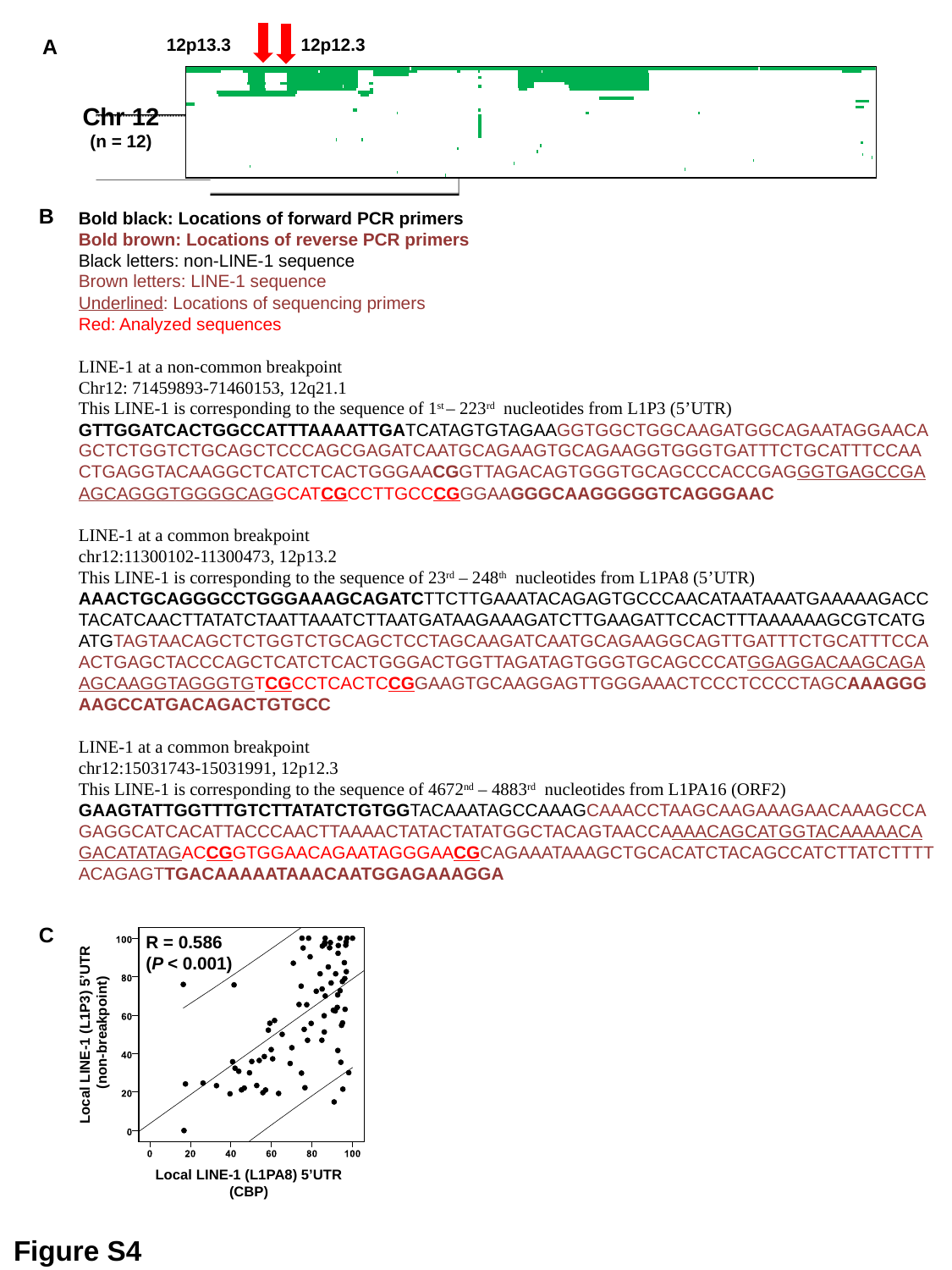

A
12p13.3
12p12.3
Chr 12
(n = 12)
B
Bold black: Locations of forward PCR primers
Bold brown: Locations of reverse PCR primers
Black letters: non-LINE-1 sequence
Brown letters: LINE-1 sequence
Underlined: Locations of sequencing primers
Red: Analyzed sequences
LINE-1 at a non-common breakpoint
Chr12: 71459893-71460153, 12q21.1
This LINE-1 is corresponding to the sequence of 1st – 223rd nucleotides from L1P3 (5’UTR)
GTTGGATCACTGGCCATTTAAAATTGATCATAGTGTAGAAGGTGGCTGGCAAGATGGCAGAATAGGAACAGCTCTGGTCTGCAGCTCCCAGCGAGATCAATGCAGAAGTGCAGAAGGTGGGTGATTTCTGCATTTCCAACTGAGGTACAAGGCTCATCTCACTGGGAACGGTTAGACAGTGGGTGCAGCCCACCGAGGGTGAGCCGAAGCAGGGTGGGGCAGGCATCGCCTTGCCCGGGAAGGGCAAGGGGGTCAGGGAAC
LINE-1 at a common breakpoint
chr12:11300102-11300473, 12p13.2
This LINE-1 is corresponding to the sequence of 23rd – 248th nucleotides from L1PA8 (5’UTR)
AAACTGCAGGGCCTGGGAAAGCAGATCTTCTTGAAATACAGAGTGCCCAACATAATAAATGAAAAAGACCTACATCAACTTATATCTAATTAAATCTTAATGATAAGAAAGATCTTGAAGATTCCACTTTAAAAAAGCGTCATGATGTAGTAACAGCTCTGGTCTGCAGCTCCTAGCAAGATCAATGCAGAAGGCAGTTGATTTCTGCATTTCCAACTGAGCTACCCAGCTCATCTCACTGGGACTGGTTAGATAGTGGGTGCAGCCCATGGAGGACAAGCAGAAGCAAGGTAGGGTGTCGCCTCACTCCGGAAGTGCAAGGAGTTGGGAAACTCCCTCCCCTAGCAAAGGGAAGCCATGACAGACTGTGCC
LINE-1 at a common breakpoint
chr12:15031743-15031991, 12p12.3
This LINE-1 is corresponding to the sequence of 4672nd – 4883rd nucleotides from L1PA16 (ORF2)
GAAGTATTGGTTTGTCTTATATCTGTGGTACAAATAGCCAAAGCAAACCTAAGCAAGAAAGAACAAAGCCAGAGGCATCACATTACCCAACTTAAAACTATACTATATGGCTACAGTAACCAAAACAGCATGGTACAAAAACAGACATATAGACCGGTGGAACAGAATAGGGAACGCAGAAATAAAGCTGCACATCTACAGCCATCTTATCTTTTACAGAGTTGACAAAAATAAACAATGGAGAAAGGA
C
R = 0.586
(P < 0.001)
Local LINE-1 (L1P3) 5’UTR
 (non-breakpoint)
Local LINE-1 (L1PA8) 5’UTR (CBP)
Figure S4
