# Supplementary material for: Genomic vulnerability to LINE-1 hypomethylation is a potential determinant of the clinicogenetic features of multiple myeloma
Source: Genome Med. 2012 Dec 22;4(12):101. doi: 10.1186/gm402 (PMC4064317; doi:10.1186/gm402)
Supplement: Additional file 8 — Figure S5. (A,B) Kaplan-Meier curves for overall survival from time of: (A) initial diagnosis for patients with MM stratified based on long interspersed nuclear element-1 (LINE-1) methylation levels; (B) sample collection for patients with MM after stratification based on the presence or absence of 13q deletion; and (C) initial diagnosis for patients with MM stratified based on the levels of methylation of the indicated repetitive elements. [file gm402-S8.PPT]

## Slide 1
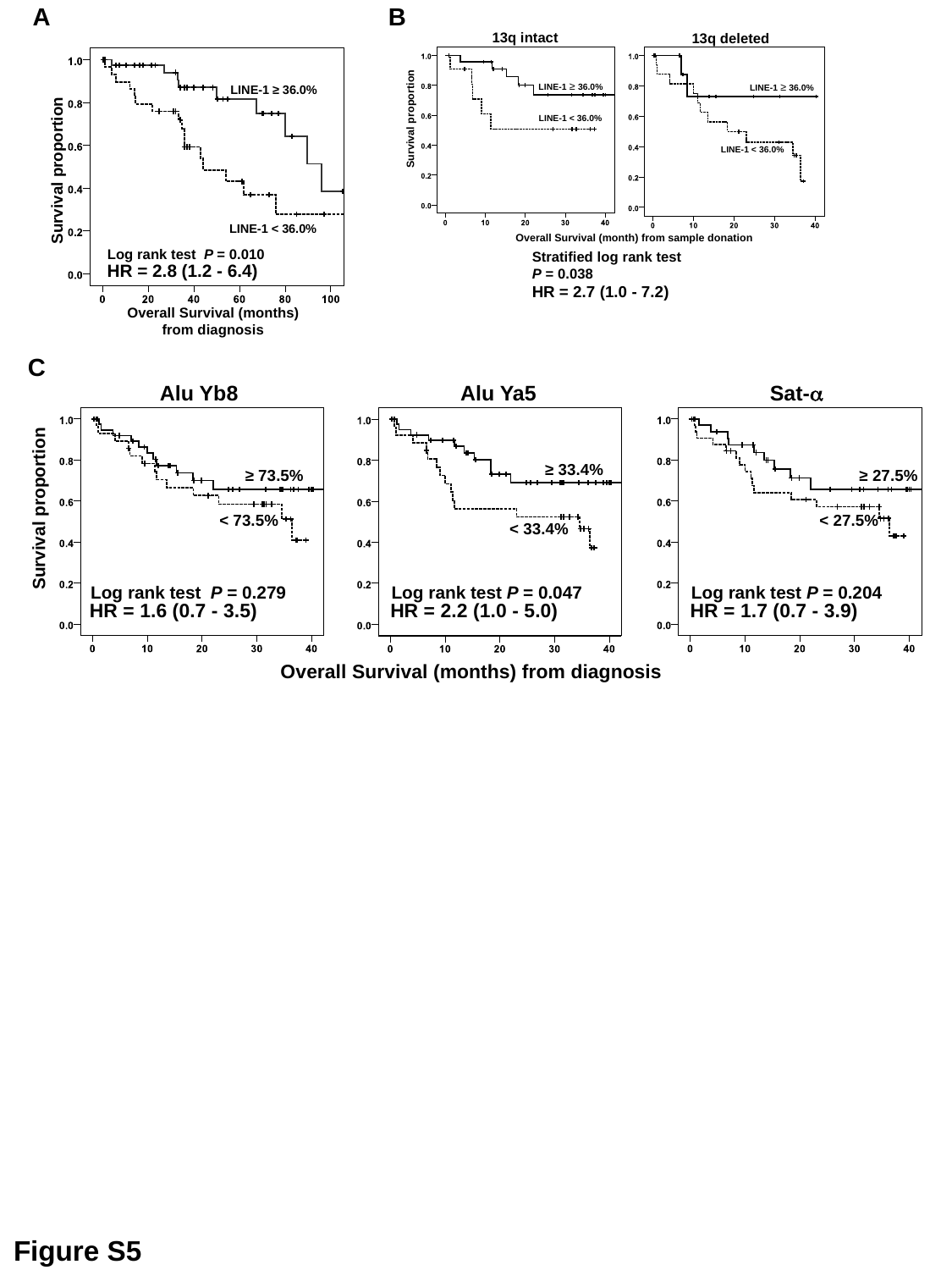

A
B
13q intact
13q deleted
LINE-1 ≥ 36.0%
LINE-1 ≥ 36.0%
LINE-1 ≥ 36.0%
Survival proportion
LINE-1 < 36.0%
LINE-1 < 36.0%
Survival proportion
LINE-1 < 36.0%
Overall Survival (month) from sample donation
Log rank test P = 0.010
Stratified log rank test
P = 0.038
HR = 2.7 (1.0 - 7.2)
HR = 2.8 (1.2 - 6.4)
Overall Survival (months)
from diagnosis
C
Alu Yb8
Alu Ya5
Sat-α
≥ 33.4%
≥ 73.5%
≥ 27.5%
Survival proportion
< 73.5%
< 27.5%
< 33.4%
Log rank test P = 0.279
Log rank test P = 0.047
Log rank test P = 0.204
HR = 1.6 (0.7 - 3.5)
HR = 2.2 (1.0 - 5.0)
HR = 1.7 (0.7 - 3.9)
Overall Survival (months) from diagnosis
Figure S5
